# The association between physical activity and depression in emerging adults: the pathway of subjective exercise experience

**DOI:** 10.3389/fpubh.2025.1718409

**Published:** 2026-01-29

**Authors:** Qi-Qi Shen, Ling-Ling Hu, Sheng-Jie Geng, Lei Cui

**Affiliations:** 1 College of P.E. and Sports, Beijing Normal University, Beijing, China; 2 Department of Physical Education, China Agricultural University, Beijing, China; 3 Student Affairs Office, China Conservatory of Music, Beijing, China

**Keywords:** depression, emerging adults, mediation, physical activity, subjective exercise experience

## Abstract

**Objective:**

Individuals during emerging adulthood, particularly college students, show a higher prevalence for sedentary behavior and its associated depression. The aim of this study is to explores depression among college students in emerging adulthood in China, investigate the association between different levels of physical activity (PA) and depression among emerging adults and examine the potential mediating role of subjective exercise experience.

**Methods:**

A total of 2,516 college students in China were selected as research subjects. The short form of an international physical activity questionnaire, a self-rating depression scale, and a subjective exercise experience scale were used to assess physical activity, depression, and exercise experience, respectively.

**Results:**

(1) In terms of depression, 921 emerging adults reported depressive symptoms (40.5%), among whom 707 had mild depression (31%) and 214 had severe depression (9%). Emerging adults with high levels of PA have significantly lower levels of depression than those with medium and low levels of PA do, and those with low levels of PA have higher levels of depression than those with medium levels of PA do. (2) In terms of subjective exercise experience, compared with those who engage in medium or low levels of PA, emerging adults with high levels of PA experience more positive (positive wellbeing, PWB) and fewer negative (psychological distress, PD; fatigue) emotions during exercise. Additionally, compared with emerging adults with lower levels of PA, those with medium levels of PA experience fewer negative emotions (fatigue). (3) Depression was negatively correlated with PA and PWB and positively correlated with PD and fatigue. PA, PWB, PD, and fatigue all served as significant predictors of depression, and their total predictive power was 45.6%. PA had a significant direct effect on depression and a significant total indirect effect through its mediators. Four significant pathways were involved, and PWB emerged as the primary mediator. The most substantial pathways included direct mediation through PWB and serial mediation through PWB and PD.

**Conclusion:**

The rate of depression among emerging adults in China is concerning. Higher levels of PA were associated with lower levels of depression; the more PA that one engaged in, the better their depression status. Both the amount of PA and the number of subjective exercise experience (PWB, PD, fatigue) were significantly associated with depression. The relationship between PA and depression involved multiple psychological pathways, which were primarily characterized by the mediating role of enhanced PWB experienced during physical activity. This highlights that future exercise interventions should target the enhancement of subjective wellbeing during activity, rather than focusing solely on increasing its volume or intensity.

## 1 Introduction

Depressive disorder (also known as depression) is a common mental disorder that involves a depressed mood or a loss of pleasure or interest in activities that lingers for long periods of time. According to data released by the World Health Organization (WHO) in 2021, 280 million people live with depression, which accounts for 3.8% of the global population (1). Beyond its direct psychological burden, depression is a leading contributor to the global disease burden and is strongly associated with increased risks of suicide and various physical health conditions, including cardiovascular disease, cancer, and diabetes (2). This highlights its status as a critical public health issue.

College students, who are in the transitional life stage between adolescence and full adulthood, represent a population of particular concern. Arnett (3) first proposed the concept of “emerging adulthood” to characterize this stage, as it specifically refers to the period ranging from later adolescence to the age of 20 years. During this stage, individual growth and development present multiple dimensions and complexities, and age is no longer the single factor that affects growth. Emerging adults face increased pressure, such as that caused by academic, professional, and interpersonal relationships, which can easily lead to emotional problems. Studies have shown that 15.32% of college students experience depression (4), and other studies have shown that the prevalence of mild to severe depressive symptoms is 35.2% (41). A recent meta-analysis of the primary mental health problems among Chinese college students covering the period from 2010 to 2020 revealed a significant increase in the incidence of depression among Chinese college students over the past decade (5). Consequently, identifying measures that can protect this population from depression is crucial.

Individuals in their emerging adulthood period, particularly college students, show a higher prevalence of sedentary behavior and its associated mental health difficulties. Physical activity (PA) is associated with a lower likelihood of depression symptoms (6). PA can both lessen the detrimental impact of anxiety on suicidal thoughts and potentially reduce the probability of suicidal ideation (7). This relationship appears to be dose-responsive. Studies have also revealed that improving emotional states can be achieved only when physical activity reaches a certain level; the longer the duration, intensity, and frequency of physical activity are, the easier the promotion of positive emotions and the reduction of negative emotions becomes (8). Recent research has further refined this understanding, indicating that low-to-moderate intensity PA may have significant protective effects against depression. This association was consistent across various age groups, sex, and geographical regions (9). Although prior evidence generally supports a negative association between PA and depressive outcomes, these relationships may not be strictly unidirectional. Depressive symptoms (e.g., anhedonia, fatigue) can reduce motivation and energy for PA, while negative subjective experiences during exercise (e.g., heightened distress) may further discourage future participation, creating a potential vicious cycle (10). Notwithstanding this potential bidirectionality, a crucial scientific and public health question persists: how can PA be most effectively harnessed to alleviate depressive mood, states, or symptoms? Thus, further research is needed to clarify both the differential effects of various PA levels on emotions and the precise psychological mechanisms that underlie these benefits, particularly among emerging adults.

While established affective response frameworks, such as the dual-mode model (11), have been used to effectively explain how exercise intensity interacts with physiological feedback to shape in-task emotional states, they are often focused on a narrow spectrum of affective valence and arousal. The experience of physical exercise, as an intuitive experience that occurs during physical activity, may be an important contributor to postexercise emotional experiences. Extending beyond purely physiological accounts, our suggestion is that subjective experience during exercise serves as a key explanatory pathway. The subjective exercise experience (SEE) framework offers a more comprehensive, multidimensional perspective (12) than traditional frameworks do. Previous research uses the SEE framework identified three exercise experience profiles among college students: positive, fatigue dominant, and negative, and showed that negative profiles relate to probable anxiety whereas fatigue dominant profiles relate to probable depression (13), further underscoring its relevance for mental health. Rather than a transient mood state, SEE reflects a structured psychological response during physical activity. Such evidence highlights SEE as a theoretically suitable pathway for understanding how PA is associated with long-term emotional outcomes. SEE encompasses not only transient psychological distress (PD) and fatigue but also incorporates a crucial cognitive-evaluative component: positive wellbeing (PWB). PWB reflects perceptions of accomplishment, mastery, and psychological renewal derived from exercise, representing a build-up of positive psychological resources rather than a fleeting feeling. This dimension captures individuals' perceptions of their own accomplishments, mastery, and the psychological refreshment that is gained from the activity itself. This broader perspective is theoretically well suited to elucidate the pathway link between PA and sustained mental health outcomes such as depression, as it accounts for both the reduction in negative states and the cultivation of positive psychological resource—a process congruent with the broaden-and-build theory of positive emotions (14). Preliminary research supports the relevance of exercise-related experiences and indicates that positive perceptions of physical activity are linked to higher levels of both PA and self-esteem (15), indicates that subjective exercise experiences may mediate the relationship between PA and depression.

Crucially, the components of SEE may function in a theoretically coherent sequence. The broaden-and-build theory (14) posits that positive affective states (like PWB) broaden cognitive-behavioral repertoires and build lasting psychological resources, which can enhance resilience and buffer against subsequent negative states like PD. Thus, PWB generated during PA may initiate the process by mitigating distress. And other studies have shown that, PD is a potent antecedent of both cognitive and physical fatigue, as sustained psychological strain depletes regulatory resources (42). Consequently, a reduction in PD following enhanced PWB may lead to decreased perceptions of fatigue. Research shows that affective responses to physical activity are multidimensional and temporally structured, with positive appraisals emerging early and psychological distress and fatigue appearing later as demands increase (16). Large-scale reviews also suggest exercise alleviates depressive symptoms partly through reducing distress and fatigue (17), but the initiating role of PWB in this sequence remains underexplored.

Despite this progress, a number of critical questions remain unanswered. First, the associations among the different levels of physical activity (low, moderate, and high), depression and the components of subjective exercise experience (PWB, PD, and fatigue) among emerging adults require clearer delineation. Second, and more critically, the potential mediating role of SEE in the link between PA and depression, particularly as it functions through sequential pathways that involve these psychological components, has not been extensively tested. Elucidating this pathway is essential for moving beyond generic PA prescriptions toward designing optimized interventions that specifically target key psychological mechanisms like PWB. Therefore, this study is aimed at exploring physical activity levels and depression detection rates among emerging adults and investigating the relationships between different levels of physical activity and depression among individuals in emerging adulthood, with a specific focus on subjective exercise experience, to elucidate how physical activity is associated with depressive symptoms. On the basis of the theoretical framework and the literature, we propose the following hypotheses:

H1: Higher levels of PA are associated with lower levels of depression.
H2: Higher levels of PA are associated with more positive subjective exercise experiences (higher PWB, lower PD and fatigue).
H3: The dimensions of SEE (PWB, PD, and fatigue) mediate the relationship between PA and depression.
H4: The mediating effect involves significant sequential pathways, specifically those from PA to increased PWB, which in turn leads to reduced PD and subsequently lower fatigue and ultimately contributes to a decrease in depression (PA → PWB → PD → fatigue → depression).

## 2 Materials and methods

### 2.1 Participants and data collection

A total of 2,516 participants in the emerging adulthood period in China completed this survey during the period from November 2023 to January 2024. A convenience sampling method was used to recruit emerging adult college students from several universities in Beijing. Recruitment was facilitated through university physical education teachers and college student social media groups, where an invitation letter that contained a survey link was distributed. This cross-sectional survey was conducted through the online survey website Wenjuanwang (http://www.wenjuan.com), which is a widely used survey site in China. The questionnaire was available online through the survey website, and the platform generated an access link for the respondents.

The inclusion criteria were (1) being between 18 and 25 years of age (the emerging adulthood period), (2) being a full-time student, and (3) having provided online informed consent. The exclusion criteria included (1) incomplete questionnaires, (2) failing to pass attention-check questions that were embedded in the survey to ensure data quality (e.g., trap questions such as “How many fingers does a human typically have?”), (3) a completion time of less than the preset minimum time, and (4) being outside of the 18–25 year age range.

Initially, 2,516 students accessed and began the survey. Written informed consent was obtained from all participants prior to their participation. This study was approved by the Academic Committee of the School of Physical Education and Sports, Beijing Normal University, with the Ethics Approval Number BNU2023-090101. After the data were screened, 243 participants were excluded on the basis of the aforementioned criteria. A total of 2,273 participants were included in the final analysis, and the response rate was 90.34%. A sensitivity power analysis was conducted using G^*^Power 3.1 to assess whether the final sample size was adequate. With a total sample size of 2,273, a two-tailed α level of 0.05, and desired power(1-β) of 0.8, the analysis indicated that our study design was sufficiently sensitive to detect a population correlation coefficient as small as |ρ| = 0.059. The analysis also provided the critical values for the sample correlation coefficient *r* under the null hypothesis. A sample correlation with an absolute value greater than |*r*| > 0.041 is required to achieve statistical significance. Because this threshold is substantially smaller than the effect sizes (β = 0.1313) observed in the present study, the available sample size can be considered sufficient for the analyses.

### 2.2 Physical activity questionnaire short form

Physical activity was measured using the validated International Physical Activity Questionnaire-Short Form (IPAQ-SF). This 7-item questionnaire is used to capture the amount of physical activity engaged in over the previous 7 days. The score was calculated according to established methods, i.e., by quantifying the physical activity metabolic equivalent (MET) min/day. The physical activity level was quantified as metabolic equivalent (MET) hours/week by multiplying the METs, duration, and frequency of exercise activities; the data are presented as METs min/day. The participants were also categorized as having low, moderate, or vigorous physical activity levels according to their METs (min/week). The validity of the IPAQ-SF for Chinese young adults aged 18–25 has been established (43), and its MET-based classification effectively discriminates the activity levels in Chinese college populations (8). Reliability data for the IPAQ short questionnaires were again deemed to be acceptable, with 75% of the correlation coefficients observed above 0.65, and the criterion validity had a median rho of approximately 0.30, which was comparable to most other self-report validation studies (18).

### 2.3 Self-rating depression scale (SDS)

Depressive symptoms were assessed according to a self-rating depression scale (19). The scale is a 20-item, self-reported index that captures the frequency of the depressive symptoms experienced within a single week. The cutoff scores for the scale were as follows: < 50 (normal), 50 to 59 (mild), 60 to 69 (moderate), and >69 (severe). The internal consistency (alpha) of the SDS was 0.92 (20). This scale has previously been applied and validated in a Chinese population (21, 22).

### 2.4 Subjective exercise experience scale (SEES)

Psychological experiences were measured via the 3-factor Subjective Exercise Experiences Scale (SEES) immediately prior to commencement and again after the completion of each class (12). The questionnaire uses a Likert-type scoring system with responses ranging from 1 (not at all) to 7 (very much so) and a midpoint anchor of 4 (moderately). The items measured included positive wellbeing (PWB), psychological distress (PD), and fatigue. The questionnaire has been validated in the Chinese population (44) and has been shown to have good reliability and validity. Its internal consistency has been reported to be high, with Cronbach's α coefficients of 0.86 and 0.85 for positive wellbeing, PWB and PD, respectively. The items pertaining to fatigue were not scored (23). The Cronbach's alpha coefficient for this scale is 0.70, indicating that it has good internal consistency reliability.

### 2.5 Statistical analysis

All the statistical analyses were performed using the Statistical Package for the Social Sciences (SPSS; SPSS Inc., Chicago, IL, USA) version 25.0 for Windows. The statistical significance threshold was set at *p* < 0.05. One-way ANOVA was used to compare depression and subjective exercise experience across clusters that were stratified by physical activity level. To investigate the relationships between physical activity and psychological outcomes, we conducted Pearson correlation and stepwise regression analyses and computed the physical activity scores, subjective exercise experience, and depression scores. A serial multiple mediation analysis using PROCESS Macro Model 6 with 5,000 bootstrap samples was conducted to examine the mechanisms linking physical activity to depression through the sequential pathways of psychological wellbeing (PWB), psychological distress (PD), and fatigue. To enhance the methodological rigor and control for potential confounders, sleep quality (PSQI total), sex, education level, and anxiety symptoms were all included as covariates in the model. Given the convenience sampling approach, the present findings should be interpreted with caution regarding generalizability beyond similar college-based emerging-adult populations, and because PA was assessed using the IPAQ-SF, which may overestimate activity levels, PA scores were treated as relative indicators of activity rather than precise objective values when interpreting associations.

## 3 Results

### 3.1 Characteristics of depression

In terms of depression, 921 emerging adults reported depressive symptoms (40.5%), among whom 707 had mild depression (31%) and 214 had severe depression (9%); 1,352 of the emerging adults reported no depressive symptoms (60%).

### 3.2 Characteristics of physical activity

A total of 2,273 emerging adults were selected as research subjects. In terms of the standard physical activity level, 652 of the emerging adults had a low level of physical activity (28.7% of the sample), 738 had a medium level of physical activity (32.5% of the sample), and 883 had a high level of physical activity (38.8% of the sample).

### 3.3 Effects of physical activity level on depression

**Figure 1:**
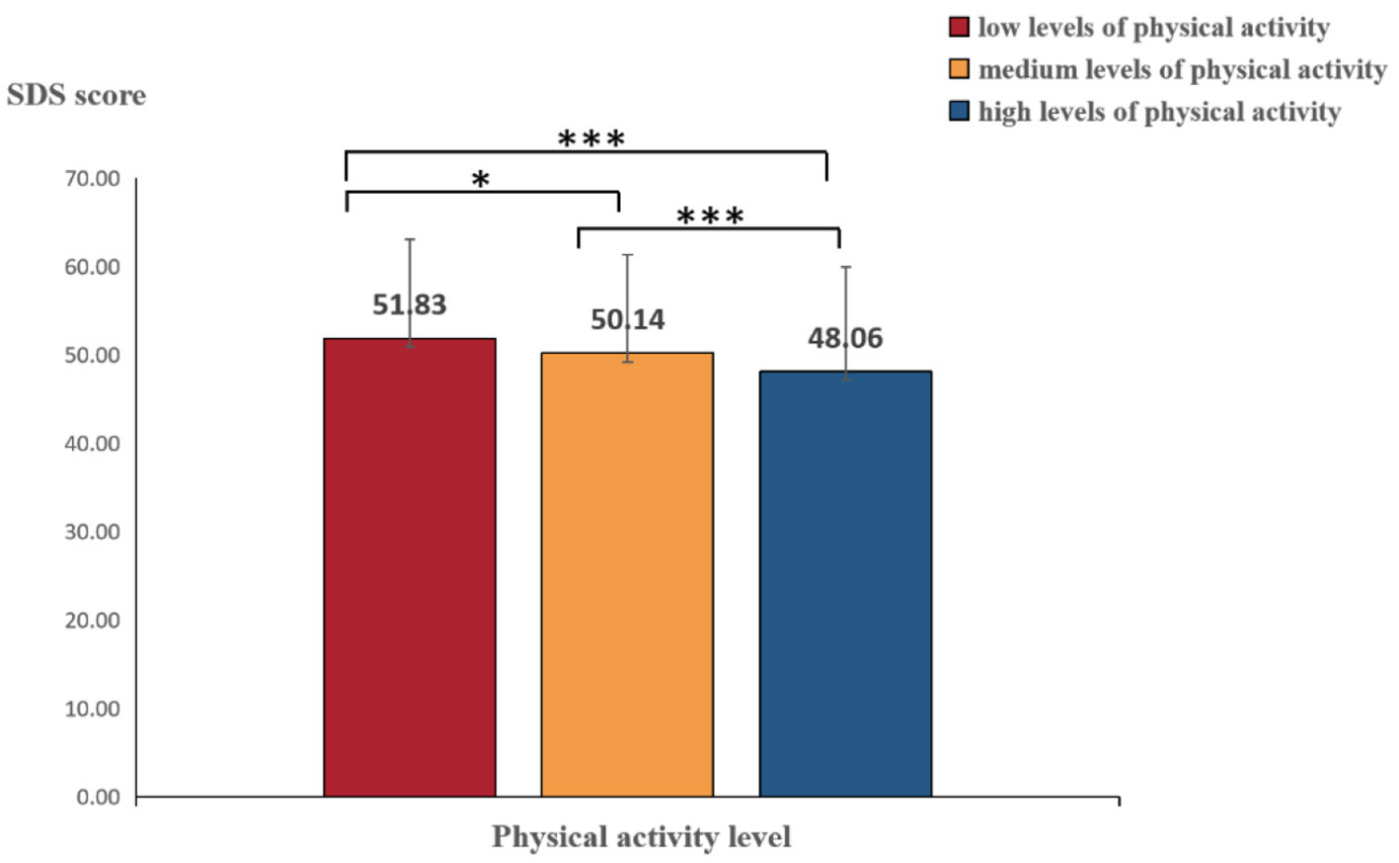
Effects of physical activity level on depression. ***Represents *p* < 0.001, and *represents *p* < 0.05.

The results revealed significant variation among the scores for depression [*F*_(2, 2270)_ = 20.802; *p* < 0.001; *partial* η^2^= 0.018]. Therefore, we further conducted *post hoc* multiple comparisons that revealed that the depression score of those emerging adults with high physical activity levels was significantly lower than that those with medium and low physical activity levels (*ps* < 0.001), and the depression score of emerging adults with medium physical activity levels was significantly lower than that of those with lower physical activity levels (*p* = 0.018 < 0.05). These findings indicate that the greater the level of physical activity was, the lower the depression state, as shown in Figure 1.

### 3.4 Effects of physical activity levels on subjective exercise experience

The results revealed that the scores for PWB [*F*_(2, 2270)_ = 26.618, *p* < 0.001; *partial* η^2^= 0.023], PD [*F*_(2, 2270)_ = 12.593, *p* < 0.001; *partial* η^2^= 0.011), and fatigue [*F*_(2, 2270)_ = 16.448, *p* < 0.001; *partial* η^2^= 0.014] differed significantly.

**Figure 2:**
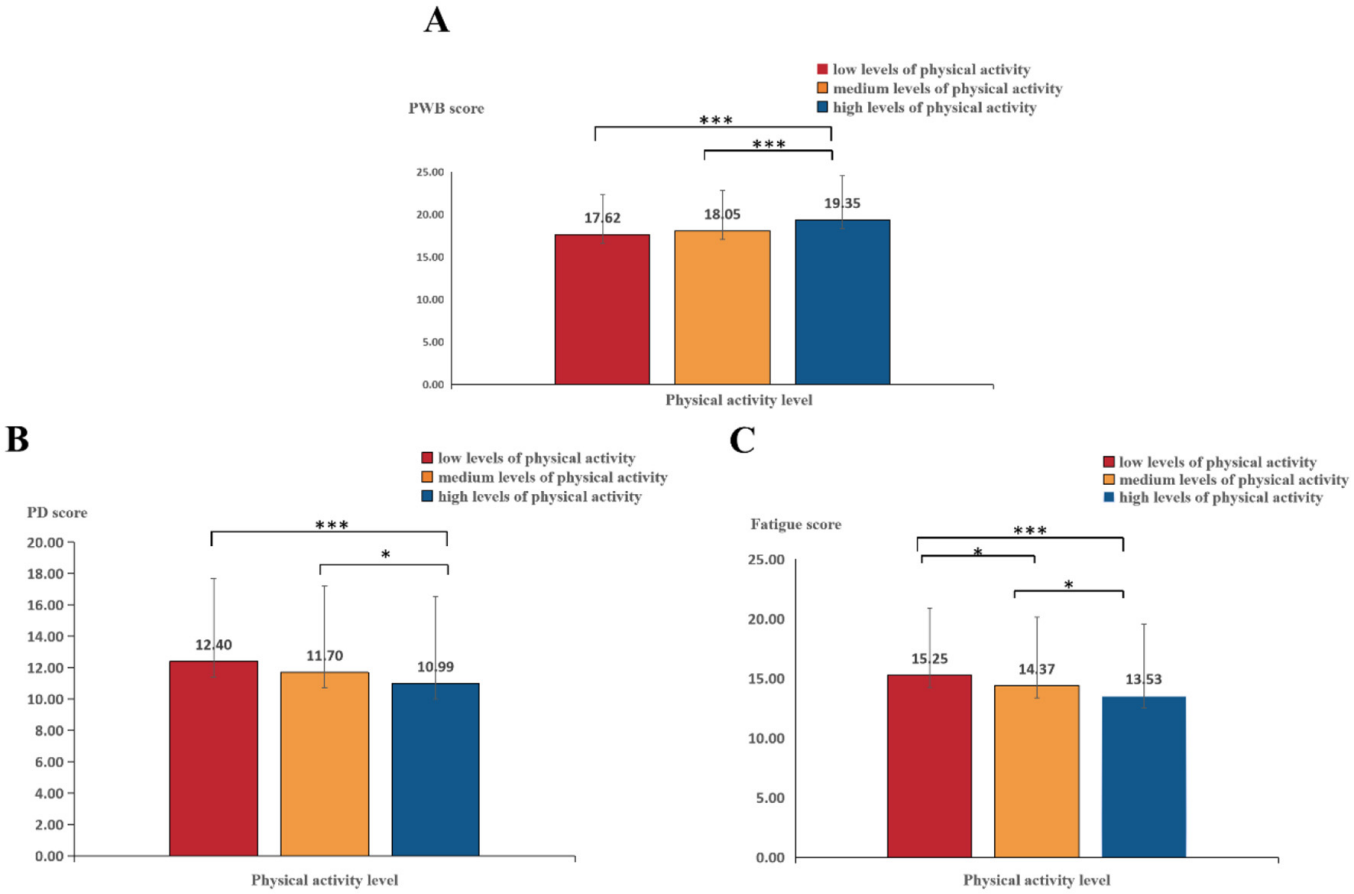
Effects of physical activity level on subjective exercise experience. **(A)** PWB. **(B)** PD. **(C)** Fatigue. ***Represents *p* < 0.001 and *represents *p* < 0.05.

Therefore, we conducted further *post hoc* multiple comparisons, which revealed that the scores for PWB among emerging adults with high physical activity levels were significantly greater than that of those with medium and low physical activity levels (*ps* < 0.001), and PD and fatigue scores among emerging adults with high (*ps* < 0.001) and medium (*ps* < 0.005) physical activity levels were significantly lower than the scores among those with low physical activity levels; moreover, the fatigue score among emerging adults with high physical activity levels was significantly lower than that among those with medium physical activity levels (*p* < 0.05). These findings indicate that the greater the level of physical activity is, the lower the negative subjective exercise experience and the greater the positive subjective exercise experience, as shown in Figure 2.

### 3.5 Analysis of the mediating effect of subjective exercise experience on the relationship between physical activity and mental health

#### 3.5.1 Correlation analysis

Physical activity was negatively correlated with depression (*r* = −0.131, *p* < 0.001), PD (*r* = −0.111, *p* < 0.001), and fatigue (*r* = −0.133, *p* < 0.001) and positively correlated with PWB (*r* = 0.152, *p* < 0.001). The depression score was negatively correlated with PWB (*r* = −0.571, *p* < 0.001) and positively correlated with PD (*r* = 0.616, *p* < 0.001) and fatigue (*r* = 0.512, *p* < 0.001).

#### 3.5.2 Stepwise regression analysis

The results revealed that physical activity, PWB, PD, and fatigue served as significant predictors of depression scores, with a total predictive power of 45.6%. The predictive power for the depression score was 0.1% for physical activity, 0.3% for the fatigue score, 7.2% for the PWB score, and 37.9% for the PD score.

#### 3.5.3 The chain mediation model

The total effect of physical activity on depression was significant (β = −0.1313; *p* < 0.001). When the mediators were included in the model, the direct effect of physical activity on depression remained significant but was substantially reduced [β = −0.0311, *p* = 0.0482; BootSE = 0.0001; 95% Boot CI (−0.0003, 0.000)], suggesting partial mediation. This attenuation indicates that approximately 76% of the total PA-depression association was accounted for by the subjective exercise experience pathways. The overall indirect effect through all proposed mediators was significant [β = −0.1002, BootSE = 0.0147, 95% BootCI (−0.1291, −0.0715)]. An examination of the specific indirect effects revealed four significant mediating pathways (Table 1):

- Simple mediation through PWB alone (Ind1)
- Simple mediation through fatigue alone (Ind3)
- Two-step serial mediation through PWB and then PD (Ind4)
- Three-step serial mediation through PWB, PD, and finally fatigue (Ind7)

**Table 1:** Direct, indirect, and total effects of physical activity on depression.

**Effect type**	**Effect (β)**	**BootSE**	**95% BootCI**
Ind1: PA → PWB → Depression	−0.048	0.008	[−0.064, −0.033]
Ind3: PA → Fatigue → Depression	−0.004	0.002	[−0.007, −0.001]
Ind4: PA → PWB → PD → Depression	−0.032	0.006	[−0.043, −0.021]
Ind7: PA → PWB → PD → Fatigue → Depression	−0.005	0.002	[−0.009, −0.002]

PA, physical activity; PWB, psychological wellbeing; PD, psychological distress; all effects are presented as standardized coefficients (β); BootSE, bootstrap standard error; BootCI, bootstrap confidence interval based on 5,000 bootstrap samples.

**Figure 3:**
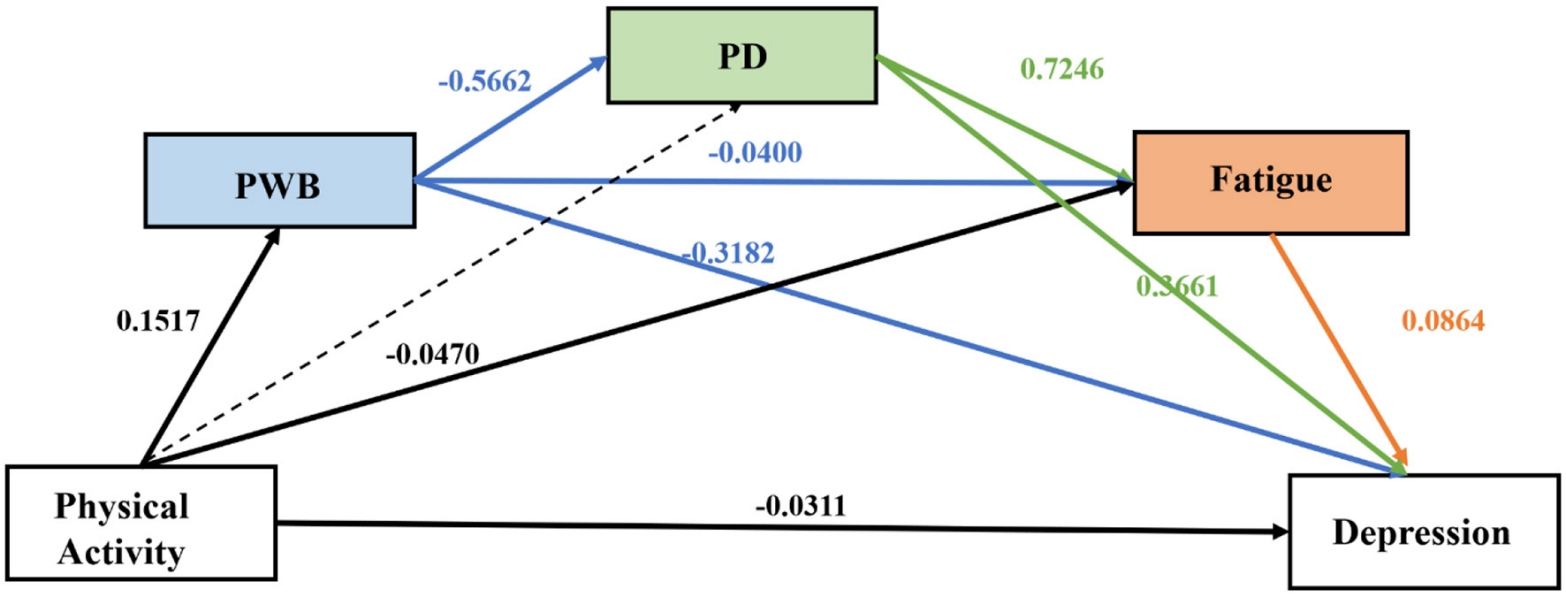
The chain mediation model. All effects are presented as standardized coefficients (β) as indicated in the figure. The dashed lines represent non-significant pathways.

These findings indicate that physical activity is associated with lower depression levels both directly and indirectly through multiple pathways, with the strongest indirect effect occurring through PWB alone, followed by the serial pathway through PWB and PD, as shown in Figure 3.

After controlling for the aforementioned covariates, the total effect of physical activity on depression was significant (β = −0.0718; *p* < 0.001), and the direct effect remained statistically significant with a reduced magnitude [β = −0.0361; *p* = 0.010; BootSE = 0.0001; 95% BootCI (−0.0003, 0.0000)], thereby indicating partial mediation. The overall indirect effect through all proposed mediators was significant [β = −0.0357, BootSE = 0.0069, 95% BootCI (−0.0492, −0.0219)].

**Figure 4:**
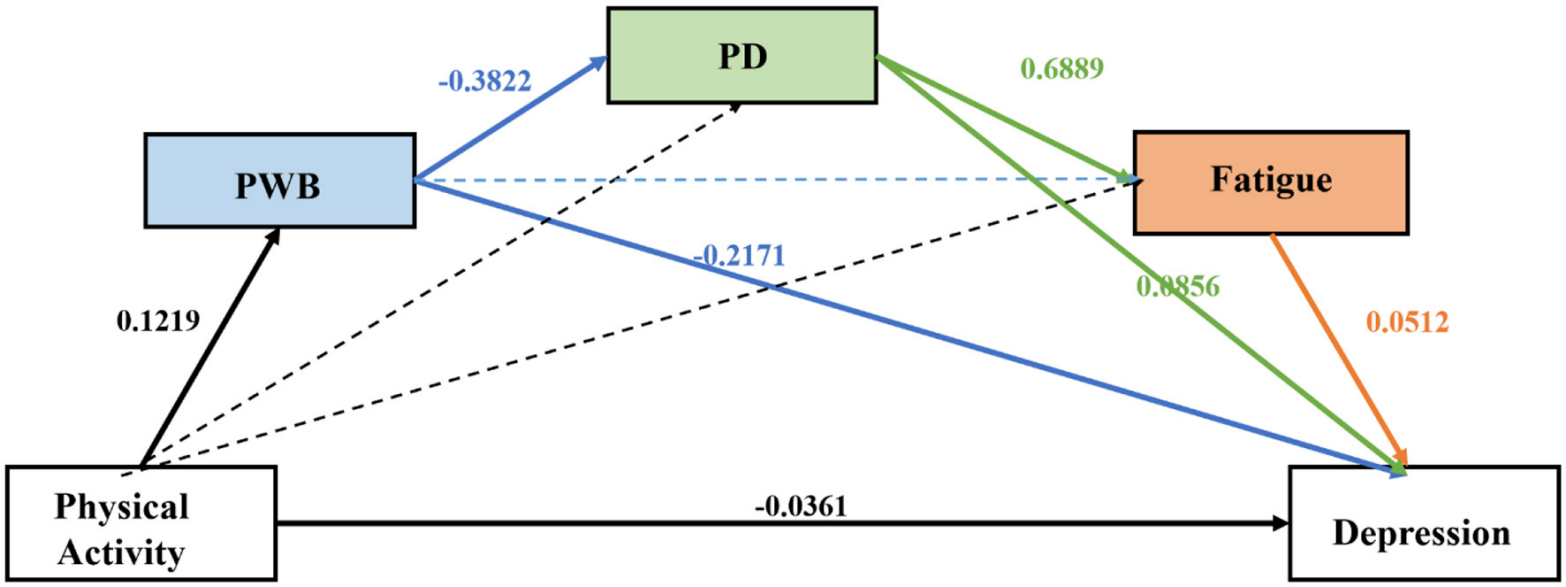
The chain mediation model. All effects are presented as standardized coefficients (β) as indicated in the figure. The dashed lines represent non-significant pathways.

Compared with the previous intermediate model (Table 1, Figure 3), after including the covariates, the path of Ind3 (the simple mediation through fatigue alone) became non-significant. An analysis of the specific indirect pathways revealed three significant mediation routes (Table 2, Figure 4):

- Simple mediation through PWB alone (Ind1)
- Two-step serial mediation through PWB and then PD (Ind4)
- Three-step serial mediation through PWB, PD, and finally fatigue (Ind7)

**Table 2:** Direct, indirect, and total effects of physical activity on depression.

**Effect type**	**Effect (β)**	**BootSE**	**95% BootCI**
Ind1: PA → PWB → Depression	−0.0265	0.0055	[−0.0375, −0.0157]
Ind4: PA → PWB → PD → Depression	−0.0040	0.0015	[−0.0072, −0.0014]
Ind7: PA → PWB → PD → Fatigue → Depression	−0.0016	0.0008	[−0.0033, −0.0003]

PA, physical activity; PWB, psychological wellbeing; PD, psychological distress; all effects are presented as standardized coefficients (β); BootSE, bootstrap standard error; BootCI, bootstrap confidence interval, which was based on 5,000 bootstrap samples.

## 4 Discussion

### 4.1 Depression levels of emerging adults

The detection rate of depression among the emerging adults surveyed in this study was 40.5% (31.1% for mild depression and 9.2% for severe depression), and this accounted for a relatively large proportion. This is consistent with the results of previous survey. The incidence of depression in the 10–24-year-old age group increased sharply across different years (40). A previous study predicting the development trend of depression among Chinese adolescents aged 10–24 years over the period of 2020 to 2029 also revealed that the annual incidence rate and DALY rate of the 20–24 age group from 1990 to 1999 were higher than those of the 15–19 and 10–14 age groups were (24). Depression among this age group is relatively severe, which deserves greater attention.

A possible reason for this is that although college students have reached adulthood in terms of physiological age, they still live in a campus environment and yet to enter society. Universities play a key role in the socialization of college students, and campuses serve as microcosms of society. Throughout this process, students must acquire interdisciplinary knowledge, develop independence, and adapt to social rules and interpersonal interactions. In this process, the inability to successfully adapt to stress can lead to pathological changes that can give rise to depression (23, 25). Students face issues such as academic and family stress (26), challenges in interpersonal relationships, and engaging in arguments and poor communication, all of which influence victimization and depressive symptoms (39).

The 40.5% prevalence identified in our study highlights the urgency of addressing mental health disparities in this age group. This rate is higher than the global adult depression prevalence of 5.7% that has been reported by the World Health Organization (27) and exceeds the 20.8% that has been observed in recent meta-analyses of Chinese college students. The research results also suggest that depressive symptoms among college students—particularly in China—may be increasing in recent years, underscoring a growing public mental health concern that warrants targeted attention and intervention. It is important to note, however, that the detection rate in the present study reflects scores derived from a self-report screening tool rather than clinical diagnostic interviews, and may therefore be influenced by response bias. In addition, the SDS categorizes mild depression (31%), severe depression only 9%; which may be transient or situational rather than meeting criteria for clinical depression, thereby contributing to a higher overall detection rate. Nevertheless, research indicates that depression remains a concerning problem. One meta-analysis covering the period of 2010–2020 revealed that the pooled prevalence of depression among mainland Chinese college students was 20.8% (5), which is particularly relevant to the 9% with severe depression who are at an elevated risk of impaired academic functioning, a reduced quality of life, and suicidal ideation (28). From a public health standpoint, this subgroup requires immediate targeted interventions (e.g., psychological counseling integrated with medical support), while the larger cohort who has mild depression represents a “window of opportunity” for the leveraging of preventive strategies, given that early intervention can mitigate symptom progression.

These findings indicate that depression remains among the leading contributors to the global disease burden and that the depression problem that occur among emerging adults is concerning. There is an urgent need for actively exploring and implementing effective prevention and treatment strategies to reduce the disease burden of depression.

### 4.2 Differing effects of different physical activity levels on mental health

This study reveals that physical activity is associated with depression. Greater levels of physical activity are associated with lesser depression. The depression scores of emerging adults with high physical activity levels were significantly better than those with medium and low physical activity levels were, and the depression scores of those with medium physical activity levels were significantly better than those with low physical activity levels were.

These findings are consistent with the results of previous research. Compared with people with low levels of physical activity, those with high levels of physical activity had lower odds of developing depression. Furthermore, physical activity has been shown to protect against the emergence of depression among both youths and adults (6). Previous studies have shown that physical activity is correlated with depression among emerging adults. Increasing the amount of physical activity is beneficial for addressing psychological problems, such as depression, among college students (29). One study involving healthy adults revealed that individuals who actively engage in PA experience positive emotions approximately 20% more often than those who do not engage in PA do (38). Notably, our study refines the understanding of the association between PA and depression by confirming significant differences across three PA intensity tiers; this granularity leads to more precise guidance for the design of stratified intervention programs.

The association between PA and depression observed in this study could be explained by several interconnected mechanisms. On the one hand, PA is linked to biological changes that can contribute to improved mood, such as the release of endogenous opioids (30), increased BDNF expression (31), and increased dopamine activity in the reward center of the brain (32). Psychologically, PA can increase self-esteem and physical health, which can potentially alleviate negative emotions (33).

However, this relationship is likely bidirectional. A previous study revealed that both fatigue and depressed mood were bidirectionally associated with physical activity. When participants perceived an increase in fatigue or depressed mood, they tended to respond by slowing down or reducing their physical activity (10). This dynamic can create a vicious cycle. Therefore, our findings are likely to reflect this complex, reciprocal interplay rather than reflecting a simple one-way effect. The higher depression scores detected in the high-PA group may represent a positive, self-reinforcing cycle, whereas the higher scores detected in the low-PA group may indicate a negative cycle.

### 4.3 Path of influence of physical activity on depression

To the best of our knowledge, this study is the first to explore the mediating role of subjective exercise experience in the relationship between physical activity and depression. This study reveals that the higher levels of physical activity were associated with more positive subjective exercise experiences (higher PWB, lower PD, and fatigue). The study further revealed a correlation between the amount of physical activity engaged in and the subjective exercise experience. This may be due to a bidirectional relationship between physical activity and exercise experience: the more frequently that physical activity is performed, the more likely that one is to experience positive feelings through exercise, and the better the exercise experience is, the more likely that one is to engage in physical activity.

This study also revealed that the amount of physical activity and subjective exercise experience (PWB, PD, and fatigue) were significantly associated with depression, and psychological distress showed the strongest association. Although the correlations between PA and the SEE variables were modest, such associations are commonly observed in behavioral health research involving large, non-clinical samples, where single behavioral predictors often account for a limited portion of variance in complex psychological outcomes (34). By contrast, the correlations among the SEE components (PWB, PD, fatigue) and depression were substantially stronger. These findings suggest that the relationship between physical activity and lower levels of psychological distress is a particularly strong component of the overall link between activity and depression. Furthermore, it is important to note that the IPAQ-SF measures general physical activity volume rather than structured exercise *per se* (18). This distinction suggests that future empirical studies are needed to specifically examine whether planned, volitional exercise exerts a differential or potentially stronger influence on these psychological pathways compared to overall daily activity.

The chain mediation model suggests that the statistical association between physical activity and depressive symptoms can be explained not by a single pathway but rather by a complex network involving the enhancement of positive psychological resources, a reduction in negative psychological states, and a decrease in fatigue. This study provides evidence for a multifaceted psychological pathway that helps explain the observed relationship between physical activity and depressive symptoms. The analytical findings indicate that when depression is considered, addressing both positive psychological resources and negative psychological states is important, as physical activity appears to be a significant factor that is related to both. PWB stands out as the most crucial mediator in the relationship between physical activity and depression. PWB not only functions as a direct mediator but also initiates sequential pathways through psychological distress and fatigue, which highlights its central role in the mental health benefits that are derived from physical activity.

Our findings elucidate the unique theoretical contribution of the SEE framework by conceptualizing subjective exercise experience as a multidimensional psychological mechanism rather than a single affective response, which extends current research on the association between physical activity and depression as well as established affective response models. Traditional frameworks, such as the dual-mode model, have been used to effectively explain how exercise intensity and interoceptive cues shape in-task affective states (11). However, the SEE framework provides a more comprehensive, multidimensional perspective. Moreover, prior literature has seldom examined how a structured set of experiences associated with exercise jointly accounts for depressive symptoms. Our study provides the first examination of a statistical sequential mediation model representing how these variables may be interrelated. The central role of PWB as the primary mediator serves as a key differentiator; PWB represents a cognitive-evaluative component that encompasses perceptions of accomplishment and mastery and extends beyond the transient physiological “feel-good” states. This finding deepens theoretical understanding of how physical activity may promote mental health and indicates that its benefits are not limited to the reduction of negative affect. This focus on the building of positive psychological resources aligns with the “broaden-and-build” theory in positive psychology (14), which suggests that physical activity can expand an individual's psychological resources, thereby building lasting resilience against depression. In summary, this study positions subjective exercise experience as a multidimensional psychological mechanism within the PA and depression model, revealing previously overlooked processes and providing new empirical grounding for a more refined framework linking PA to mental health. Notably, the relationship between the independent variable and depression is partially mediated by a sequential pathway that moves through positive affect, psychological distress, and fatigue. Positive affect serves as a crucial initial mediator in this psychological mechanism. The results indicate that the effect of the IV operates primarily through the enhancement of positive affect, which subsequently reduces psychological distress and, in turn, lessens fatigue, which ultimately leads to lower depression levels. The non-significant direct pathway from the IV to fatigue (Ind3) underscores the fact that fatigue reduction is not an independent mechanism but rather a downstream consequence of improved affect and reduced distress. This is consistent with previous research. The subjective exercise experience also influenced affective state in that it was a significant predictor of affective state changes (35). Affective–Reflective Theory (ART) exercise-related stimuli elicit rapid affective valuations that subsequently inform reflective evaluations and shape later emotional states (36). This affective mechanism complements motivational perspectives suggested by Self-Determination Theory (SDT), which further clarify how positive exercise experiences translate into emotional benefits. From the perspective of self-determination theory, positive exercise-related experiences such as PWB can be understood as indicators of competence and autonomy satisfaction during physical activity. Satisfaction of these basic psychological needs has been shown to foster vitality and emotional stability, which in turn reduces vulnerability to negative psychological states including distress and fatigue (37). These perspectives provide a theoretical rationale for why PWB emerges as the initial mediator in the sequential pathway and precedes reductions in psychological distress and fatigue in our model. It also suggests that interventions should consider strategies to enhance the PWB during exercise, not just increase volume or intensity.

Despite its contributions, several limitations of this study must be acknowledged. Given the cross-sectional design, these findings should be interpreted as correlational and warrant longitudinal or experimental confirmation. Participants were recruited from China based convenience sample, which may limit broader generalizability. Our reliance on self-report measures, including the IPAQ-SF, and SDS, introduces potential biases. PA, depression, and exercise experiences were all assessed via self-report, which may introduce recall or reporting bias. Therefore, data derived from self-report measures, including IPAQ SF scores and the SDS based depression detection rate, should be interpreted with caution. The small effect sizes could reflect unmeasured confounders (e.g., genetic predispositions and diet) or result from the measurement limitations of self-reported PA. Future studies using objective PA monitoring and longitudinal designs are needed to clarify the causal magnitudes. Moreover, the proposed sequential mediation, particularly that following the three-step pathway (PA → PWB → PD → Fatigue), remains exploratory and requires further empirical validation. Future studies should also test the cross-cultural applicability of our model among diverse populations and examine whether the primacy of PWB holds across different cultural and educational contexts.

## 5 Conclusions

The rate of depression among emerging adults in China is concerning. Higher levels of PA were associated with lower levels of depression; the more PA that one engaged in, the better their depression status. Both the amount of PA and the number of subjective exercise experience (PWB, PD, fatigue) were significantly associated with depression. The relationship between PA and depression involved multiple psychological pathways, which were primarily characterized by the mediating role of enhanced PWB experienced during physical activity. This highlights that future exercise interventions should target the enhancement of subjective wellbeing during activity, rather than focusing solely on increasing its volume or intensity.

## Data Availability

The raw data supporting the conclusions of this article will be made available by the authors, without undue reservation.
